# Microbial dynamics and biodiversity in table olive fermentation: culture-dependent and -independent approaches

**DOI:** 10.3389/fmicb.2012.00245

**Published:** 2012-07-06

**Authors:** Cristian Botta, Luca Cocolin

**Affiliations:** Department of Valorisation and Exploitation of Agroforestry Resources, Agricultural Microbiology and Food Technology Sector, University of Turin,Grugliasco, Italy

**Keywords:** table olives, fermentation, ecology, molecular methods

## Abstract

The microbial ecology of the table olive fermentation process is a complex set of dynamics in which the roles of the lactic acid bacteria (LAB) and yeast populations are closely related, and this synergism is of fundamental importance to obtain high quality products. Several studies on the ecology of table olives, both in spontaneous fermentations and in inoculated ones, have focused on the identification and characterization of yeasts, as they play a key role in the definition of the final organoleptic profiles through the production of volatile compounds. Moreover, these are able to promote the growth of LAB, which is responsible for the stabilization of the final product through the acidification activity and the inhibition of the growth of pathogenic bacteria. The current empirical production process of table olives could be improved through the development of mixed starter cultures. These can only be developed after a deep study of the population dynamics of yeasts and LAB by means of molecular methods. Until now, most studies have exploited culture-dependent approaches to define the natural microbiota of brine and olives. These approaches have identified two main species of LAB, namely *Lactobacillus plantarum* and *L. pentosus*, while, as far as yeasts are concerned, the most frequently isolated genera are *Candida*, *Pichia*, and *Saccharomyces*. However, there are a few studies in literature in which a culture-independent approach has been employed. This review summarizes the state of the art of the microbial ecology of table olive fermentations and it focuses on the different approaches and molecular methods that have been applied.

## INTRODUCTION

Table olives are the most important fermented vegetables on the international food market, with a production of almost two million tons per year. The European Union (EU), with 1.4 million tons (average production over the last 5 years), is the main producing area in the world. Spain is the leading producing country with a share of 72.8% and it is followed by Greece and Italy, which produce 15.2 and 9.6% of the table olives marketed in the EU, respectively. In Italy, 44% of the table olives is produced in Sicily and the per capita yearly consumption is about 3 kg, one-third of the domestic production (UNAPROL, 2009). Other significant non-EU producing countries include Turkey (16.4% of the world production), Egypt (13.5%), Syria (6.3%), Argentina (6.4%), and Algeria (4.7%; IOOC, 2011). In this context, the unified qualitative standard applied to table olives pertaining to international trade, defines this product as: “the sound fruit of specific varieties of cultivated olive trees (*Olea europea sativa*) harvested at the proper stage of ripeness and [...] processed as specified in this standard [IOOC, 2008]. Such processing may include the addition of various products or spices of good quality” (Segovia Bravo et al., 2007; Aponte et al., 2010, 2012).

Olive fruit is a drupe that contains a bitter component (oleuropein), a low concentration of sugar (2.6–6.0%) and a high oil content (12–30%), although these values can change according to the degree of maturity and the olive variety. Such characteristics prevent olives from being consumed directly after harvesting and several treatments have been promoted to make them more readily eatable. These treatments differ considerably from region to region (Arroyo-López et al., 2008). The most common industrial preparations are the Spanish (or “Sevillian”) style, with about 60% of the production, untreated or natural black olives (also known as Greek style) and the Californian style (Panagou et al., 2008). The first method consists of treating the fruit with a diluted NaOH solution (2–3%). The olives are then placed under brine (initial concentration of 8–12%), where they undergo lactic acid fermentation (Garrido-Fernández et al., 1997). Natural or untreated olives (green or naturally black) are instead brined directly after picking. The drupes in the brine undergo mixed-acid fermentation until they, at least partially, lose their bitterness. The fermentation period, therefore, depends on the physic-chemical conditions, that is, the cultivar, the salt content, and the temperature (Tassou et al., 2002; Arroyo-López et al., 2008; Panagou et al., 2008). In the Californian style method, ripe olives are first preserved in an aqueous solution and then treated throughout the year with a NaOH solution (alkali oxidation; Arroyo-López et al., 2008). A complete overview of the different ways of processing table olives and a complete description of olives characteristics can be found in Garrido-Fernández et al. (1997), 2006 and García-García et al. (2006).

The spontaneous fermentation that occurs in the different table olive processes are usually the result of the competitive activities of the native microbiota, together with a variety of contaminating microorganisms from fermentation vessels, pipelines, pumps, and other devices in contact with the olives and brine (Panagou et al., 2003). Therefore, table olive fermentation processes are a complex microbial ecosystem in which both competition and a slight synergism occur between the lactic acid bacteria (LAB) and yeast populations until the end of fermentation (Arroyo-López et al., 2008). Furthermore, some Gram-negative species of bacteria (mainly Enterobacteriaceae) can be found in considerable numbers at the beginning of the fermentation process. In normal conditions they decrease after the first few weeks of fermentation due to the acidification process (Panagou et al., 2003; Abriouel et al., 2011). However, if these spoilage bacteria are present in high numbers they can deteriorate the final product, due to the production of off-flavors and gas pockets on the surface of the olives (Garrido-Fernández et al., 1997). LAB are necessary to decrease the pH of brines, and to stabilize the final product in which they are the predominant microbial population, especially in the Spanish style method. Instead, in the fermentation carried out without a prior lye treatment (such as in the Greek and Californian methods) the lower presence of reducing sugars in the brine, as well as other nutrients, including vitamins and amino acids released from olive flesh, limits the development of LAB. Vice versa, the diffusion of phenolic in brine from drupe compounds can partially inhibit the growth of LAB, while the yeast population does not seem to be influenced (Tassou et al., 2002; Bautista-Gallego et al., 2011).

These factors, associated with a relatively low temperature (under 18°C) and high salt concentration (over 8%), can promote a greater development of yeasts than LAB, even in fermentation processes with initial debittering (Tassou et al., 2002; Lopez et al., 2005; Arroyo-López et al., 2008). Yeasts can dominate the microbiota during the fermentation of directly brined olives (Bautista-Gallego et al., 2011), but an excessive growth of yeast usually determines a final product with a milder taste and less self-preservation due to the high pH (Panagou et al., 2008). However, one of the beneficial aspects of yeasts is that they can produce volatile compounds, which play an important role in flavor generation and texture maintenance during fermentation and storage of the product (Arroyo-López et al., 2008).

The production of table olives in many industries has so far been conducted in an empirical way and this makes the standardization of the product particularly difficult, since the initiation of a spontaneous fermentation process takes a relatively long time (24–48 h), and there is a high risk of spoilage. During the early phases, which are associated with the lag phase of microbial growth (especially for LAB), the contaminating microorganisms increase in number and compete for nutrients. In most cases, as frequently occurs in the dairy industry for whey, brines from previous successful fermentations are added to initiate a new process. However, this method (named black-slopping) involves a critical factor, because the lactic cultures may exhibit different metabolic activities which can lead to different behavior in the production of flavor and other quality characteristics. Therefore, inoculation with pure or mixed LAB starter cultures is the main way of improving and making the fermentation process of table olives more predictable, especially in the early phases. Some commercial starter cultures of *Lactobacillus pentosus* or *L. plantarum* that are suitable for fermented vegetables in brine, such as table olives, are already on the market (Panagou et al., 2003).

There are many recent studies in the literature regarding the selection and subsequent evaluation, at a pilot scale, of autochthonous LAB strains as starter cultures (Hurtado et al., 2008, 2010; Bevilacqua et al., 2010; Aponte et al., 2012) and the utilization of probiotic strains of LAB (isolated from olives or from other environments) as starters (Bevilacqua et al., 2010; De Bellis et al., 2010). The attention of many researchers is also currently focused on the study of yeast dynamics in order to develop some yeast strains that could be used, alone or in combination with LAB, during olive processing as starters (Bevilacqua et al., 2009; Hurtado et al., 2010; Nisiotou et al., 2010; Bautista-Gallego et al., 2011).

The development of both LAB strains and mixed cultures of yeasts and LAB as starters cannot be separated from the previous detailed studies on native microbial ecology. Understanding the microbial biodiversity and dynamics of table olives is a fundamental step toward developing future native starters that will be able to control and lead the fermentation process while maintaining the original organoleptic characteristics of the product. The results of the most recent studies regarding LAB and yeast population ecology and dynamics are summarized in this review with particular attention to the different molecular approaches that have been used.

## LAB IN TABLE OLIVE FERMENTATION

As described in numerous studies, the species of LAB encountered most frequently during table olive fermentation are *L. plantarum*, *L. pentosus*, and, to a lesser extent, *L. paraplantarum* (*L. plantarum* group), both in the fermentation carried out on debittering drupes with NaOH and in directly brined fermentation (Campaniello et al., 2005; Hurtado et al., 2008; Bevilacqua et al., 2010). In natural olive fermentation (without initial inoculums) these species are usually detected by means of cultivation methods at significant loads after at least 10 days from the beginning of the technological process (Tassou et al., 2002). These species are facultative heterofermentative rods and their shift from homo- to heterofermentative metabolism are favored under environmental stress conditions of such as oxygen and nutrient limitation, salt concentration, and low pH levels (Bobillo and Marshall, 1992). In this context, Hurtado et al. (2008) noted an increased production of acetic acid in black olive fermentation inoculated with *L. plantarum* compared to *L. pentosus* inoculated fermentation. This seems to highlight a lower capacity of the *L. pentosus* strain to maintain a homofermentative metabolism under stress conditions.

In a following study, the same authors noted that in the case of the co-inoculation of strains of *L. pentosus* and *L. plantarum* only the first dominated, while the *L. plantarum* starter cultures failed to colonize the brine of green table olives (Hurtado et al., 2010). Better performances of *L. pentosus* starter strains than *L. plantarum* were also detected when they were inoculated separately as starter cultures in black olive fermentations (Panagou et al., 2008) and in Spanish style green table olives (Panagou and Tassou, 2006). Regarding the salt concentration tolerance of *L. pentosus* strains used as starters, Panagou et al. (2003) emphasized an initial decrease in inoculated LAB at the beginning of the fermentation process. This decrease is probably due to an initial adaptation of the inoculated lactobacilli to a high salt concentration. This behavior of the inoculum was not observed during black olive fermentations of either the *L. pentosus* or *L.plantarum* strains (Panagou et al., 2008).

Only few studies identified strains of LAB belonging to *L. paraplantarum*, both in Spanish style and in Greek style fermentation processes. Hurtado et al. (2008) reported a limited presence of *L. paraplantarum* strains from the third week of fermentation of untreated green table olives named *Arbequina*, while Franzetti et al. (2011) reported a minimal presence of *L. paraplantarum* in Italian marketed table olives. The *L. plantarum* and *L. pentosus* species are not only responsible for the acidification of brine during fermentation. In fact, *L. pentosus* presents a noticeable β-glucosidase activity and contributes, with the yeasts, to the biological olives debittering process (Franzetti et al., 2011). In this context, Servili et al. (2006) selected a strain of *L. pentosus* that is able to decrease the concentration of bitter compounds in olives in the first 8 days of fermentation. Landete et al. (2008) also noted this property in several strains of *L. plantarum*, a flexible species which is usually abundant in the fermentation of plant-derived raw materials, in which phenolic compounds are present in high concentrations. This activity consists of the enzymatic degradation of oleuropein and the consequent release of glucose plus aglycone and the conversion of the latter, in turn, to simpler non-bitter compounds such as elenolic acid and hydroxytyrosol. The latter is the most abundant phenolic compound present in the brine of fermented olives and it is able to inhibit the growth of LAB, but not at the concentrations usually detected in table olive brines (Marsilio and Lanza, 1998; Landete et al., 2008). The *L. plantarum* growth inhibition, mechanism due to oleuropein hydrolysis products is not yet clear. Some authors have suggested that they induce leakage of glutamate and inorganic phosphate from the bacterial cell as well as the degradation of the cell wall itself. However, it has been shown that the bactericidal effect of phenolic compounds is related to alterations of the cellular structure at two different levels: the cell wall and the cytoplasmic membrane. In this regard, it has also been described that, in the presence of high concentration of oleuropein, the typical road form of *L. plantarum* and also the typical Gram-positive appearance are lost and a Gram-negative profile has been observed under the microscope (Rodriguez et al., 2009). Marsilio and Lanza (1998) emphasized that the inhibitory effects of phenolic compounds on lactobacilli are more significant in a greater presence of NaCl. The same authors reported that the presence of *L. plantarum* is greater in low concentrate synthetic brines supplemented with oleuropein than in those supplemented with hydroxytyrosol, probably because the glucose released from the hydrolyzed glucoside is readily metabolized by the bacteria. This aspect underlines the usefulness of oleuropein as a nutrient source and not only as inhibitory compound.

In light of these studies, it can be stated that, in the fermentation of olives not treated with NaOH, the limited presence of LAB, in comparison to Spanish style fermentations, is not due mainly to the high presence of polyphenols, but to the low presence of nutrients and the different conditions of the process, such as the lower temperature. Lower temperatures reduce the ability of LAB to use the nutrient that leaks from the drupes through the pericarp, which becomes available for yeast and non-fermentative Gram-negative bacteria. The latter may be responsible for an alkalinization of the brines which can prime the proliferation of spoilage and pathogenic bacteria (Abriouel et al., 2011). In this way, the initial debittering practice may promote LAB development and increase the nutrient presence in brine, but only if the fermentation is carried out at ambient temperature. At low temperatures, the initial debittering treatment with NaOH could damage the quality and the safety of the final product because of the growth of undesired microorganisms.

The resistance to polyphenols, as well as the ability to grow in a high salt concentration matrix and at a low temperature, are prominent characteristics of those strains of LAB that are suitable for use as starters in olive fermentations. Aponte et al. (2012) isolated a considerable number of *L. coryniformis* strains from Sicilian green table olives produced according to the Spanish method. During physiological tests, these strains showed a higher resistance to phenolic compounds and β-glucosidase activity greater than *L.pentosus* strains isolated from the same fermentations. Moreover, it is interesting to note that the strains belonging to the *L. coryniformis* species were only found in samples from non-irrigated fields. This species has recently been isolated in low percentages in both green table olive fermentation (De Bellis et al., 2010) and in black olives packed in modified atmosphere conditions (Doulgeraki et al., 2012).

Another study, carried out on the same type of olives showed a strong predominance of *L. casei* (Randazzo et al., 2004). The results of this work constitute a new overview the microbiota of Sicilian table olives that have not been found in other studies. Over 50% of the LAB identified in olives collected from different areas in Sicily belonged to this species, which was followed by *L. brevis*. Recently, a study performed on a safety evaluation of loose green table olives marketed in Italy, while confirming the high presence of the *L. plantarum* group, also showed a noticeable presence of *L. casei* (Franzetti et al., 2011). The same study, with regard to LAB microbiota, reported that the dominant forms were cocci belonging to *Pediococcus* *acidilactici*, *Pediococcus parvulus*, and *Enterococcus* *faecalis*. The presence of the *Pediococcus* genus has also been detected in black olives packed in a modified atmosphere during storage. *Pediococcus ethanolidurans* was the predominant LAB species in pouches with an initial presence of O_2_. In this context, the yeast population on the olive surface may prevail and initial gas concentration is changed and the presence of CO_2_ is increased due to their respiratory activity (Doulgeraki et al., 2012). Moreover, through the production of ethanol, yeasts may cause a selection of the microbiota in favor of more ethanol-resistant species, such as *Pediococcus ethanolidurans* (Liu et al., 2006). Regarding the other LAB in olive ecology, the presence of *Leuconostoc mesenteroides* (Abriouel et al., 2011; Franzetti et al., 2011) and *Leuconostoc pseudomesenteroides *(Ercolini et al., 2006; De Bellis et al., 2010) should not be overlooked. The occurrence of these heterofermentative cocci is high, especially in fermentations carried out in brine with a low salt concentration (De Bellis et al., 2010).

## YEASTS IN TABLE OLIVE FERMENTATION

The yeast population ecology of fermented olives has already been studied in depth in numerous papers that have simultaneously investigated their technological potentials and spoilage characteristics. Olive microbiota is very heterogeneous during the fermentation period in relation to the change in the biochemical conditions of brine, such an increase in pH and organic acids and a variation of the salt concentration. Obviously, the presence of yeasts is also closely related to the raw material (ripe or green olives) and linked to the different artisanal and industrial fermentation processes that are used.

In relation to fermentation technologies, Bautista-Gallego et al. (2011) noted greater yeast counts in directly brined fermentation than in Spanish style fermented olives, in industrial settings, and observed a higher genetic biodiversity in the latter. In this context Arroyo-López et al. (2006) reported that the initial microbiota in Spanish style processed olives, is normally destroyed when heavy lye treatments are applied, whereas Bevilacqua et al. (2009) emphasized that yeast can survive NaOH processing and colonize olives throughout the fermentation. The resistance to alkaline pHs is a strain-specific characteristic, therefore not all autochthonous yeast strains are able to overcome lye treatments. As far as the two different growth matrices (brines and olives) are concerned, Hernández et al. (2007) found a greater presence of yeast cells in the brine than on the olive surfaces and also a different predominant species. Strains belonging to the *Wickerhamomyces* *anomalus* (previously known as *Pichia anomala*) species where in fact detected with a high frequency in brine, while the main species identified on drupe surface was *Cryptococcus laurentii*, and this was followed by different species of *Candida* and *Pichia* genera.

Thus, the yeast present on the surface of fresh olive fruit is generally limited (under 3 log_10_cfu g^-^^1^) and the dominant species are not the same ones that prevail in brine during the fermentation period (Hernández et al., 2007; Nisiotou et al., 2010). Moreover, the yeast community that originates from the initial microbiota on the olive surface, and which is present in the early fermentation phase, is characterized by a large number of species that do not seem to be involved in the fermentation proceedings (Arroyo-López et al., 2008; Nisiotou et al., 2010; Alves et al., 2012). Hurtado et al. (2008) and Aponte et al. (2010) have reported a high initial presence of *Candida diddensiae* and *Rhodotorula glutinis* in the early phases of *Arbequina* table olive fermentation (green olives brined directly), whereas *Pichia* *kluyveri*, together with *Candida boidinii* were the dominant microbiota species. Later, when the LAB counts overcome the yeast populations, *Pichia membranifaciens* and *Kluyveromyces lactis* appear and they remain until the end of the fermentation. Another study carried out on Spanish style green table olives showed similar behavior of the yeast populations, with an initial presence of *Candida diddensiae* and subsequently a dominance of *Pichia kluyveri* during the fermentation. These results were obtained with both culture-independent and classical microbiological techniques (Muccilli et al., 2011). As far as *Candida diddensiae* is concerned, the occurrence of this species has been reported with a high frequency in several works, but always in the early phases of fermentation (the first week) together with other species of genus *Candida* and more rarely with *S. cerevisiae* in green table olives fermented without a prior debittering treatment (Hurtado et al., 2008; Bautista-Gallego et al., 2011). *Candida diddensiae* seems to be one of the yeast species responsible for the fall in the pH at the beginning of the process. In general, it is described as a strong fermentative yeast, well adapted to the first fermentation stage (Hurtado et al., 2008). Therefore it has already been co-inoculated, together with LAB strains, and it determines an evident inhibition against spoilage bacteria such as Enterobacteriaceae (Hurtado et al., 2010). Regarding this species, Muccilli et al. (2011) reported the absence of *Candida diddensiae* in the fermentation of green table olives previously treated against olive fruit fly in the field with kaolin. This is probably due to the origin of this yeast, which is present on the carposphere of olive fruit. *Aureobasidium pullulans* and *R. glutinis* are the other species that are mainly involved at the beginning of the fermentation process (Arroyo-López et al., 2008; Bautista-Gallego et al., 2011; Alves et al., 2012). The first is a yeast like fungus with a high salt tolerance (over 17% w/v) which has recently been detected in green and black olives not treated with NaOH (Nisiotou et al., 2010; Abriouel et al., 2011; Alves et al., 2012). *R. glutinis*, and more generally the species of the *Rhodotorula* genus are of considerable concern in the elaboration of olives, as they are known to cause softening of the olive tissue because of polygalacturonase excretion and pellicle formation in brine (Nisiotou et al., 2010).

As the fermentation process progresses other species takeover from those described above. In general, *W. anomalus*, *S. cerevisiae*, *Pichia kluyveri*, *Issatchenkia orientalis* (the anamorphic state of *Candida krusei*) and *Pichia membranifaciens* are the main species isolated from different olive preparations and they are involved until the end of the fermentation process (Bevilacqua et al., 2009; Aponte et al., 2010; Nisiotou et al., 2010; Bautista-Gallego et al., 2011; Alves et al., 2012). *W. anomalus* has been detected in several olive fermentations and Hernández et al. (2007) have reported the relevance of this species in table olive fermentation as a starter culture. This species is in fact well adapted to the environmental conditions that govern table olive fermentations (low pH and high NaCl concentration) and has shown a strong β-glucosidase and esterase activity, both in quantitative and qualitative assays. Moreover, the strains belonging to this species have shown in laboratory conditions, a high production of bioactive antioxidants, that are able to retard the oxidative degeneration of fatty substances and improve human health. Furthermore, *W. anomalus* isolates from olive brines are able to produce killer toxins endowed with a broad spectrum of activity against human pathogens and spoilage microorganisms of table olives. This latter activity has been confirmed by Hernández et al. (2008) for this species and for other yeasts isolated from seasoned green table olives such as *K. marxianus*, *Pichia guilliermondii*, and *S. cerevisiae*. *S. cerevisiae* has been associated with olive fermentation since the initial scientific studies on this product. Its high occurrence during storage is consistent with the results obtained by Hernández et al. (2007) and Rodríguez-Gómez et al. (2010) for directly brined green table olives, although other authors have reported lower proportions in diverse brine solutions (Nisiotou et al., 2010). Abriouel et al. (2011) have reported the limited growth of this species in green table olive fermentations carried out with an initial addition of acetic acid. The inhibition of *S. cerevisiae* is probably due to the high lactic acid produced by LAB during this kind of fermentation. However, there is evidence that *S. cerevisiae* improves *L.pentosus* performances in green table olives (Segovia Bravo et al., 2007). *I. orientalis* is a common species that is found in fermented olives and its frequent occurrence is probably due to its multiple-stress-tolerant state, as it is acid, ethanol, salt and thermo tolerant (Muccilli et al., 2011). *Pichia kluyveri* is the main species involved in the fermentation of directly brined green table olives (Hurtado et al., 2008; Aponte et al., 2010). This species is a welcome occurrence in green table olive fermentation, because it is not able to reduce sodium lactate or sodium citrate and therefore does not change the final acidity of the product. Furthermore, the strains belonging to this species have shown considerable β-glucosidase activity, which is extremely useful to reduce the bitterness of untreated olive fruit during fermentation (Aponte et al., 2010). Finally, *Pichia membranifaciens* has been detected with a high frequency at the end of the fermentation process in loose table olives marketed in Italy and in Greek black olives packed under a modified atmosphere (Franzetti et al., 2011; Doulgeraki et al., 2012). Nevertheless, their occurrence has been reported in both Spanish style and directly brined olives, until the end of fermentation (Garrido-Fernández et al., 1997).

As previously mentioned, during table olive fermentation yeasts are involved in the production of the compounds and organic acids that are essential to define the final flavor, but they also have a significant ability to synthesize substances such as vitamins, amino acids, and purines, or breakdown complex carbohydrates, which is essential for the growth of *Lactobacillus* species as they need many nutrient sources for an optimal growth in a poor substrate such as brines (Arroyo-López et al., 2008). The co-inoculation of yeasts and LAB strains has led to an improvement in the growth of the latter and consequently in the production of lactic acid due to the greater availability of the previously listed nutrients (Segovia Bravo et al., 2007; Aponte et al., 2012).

The presence of volatile compounds, such as ethanol, glycerol, higher alcohols, and, to a lesser extent, acetaldehyde in brines can be directly attributed to the metabolic activity of yeasts together with heterofermentative bacteria (Arroyo-López et al., 2008). Instead, the presence of methanol, which is often detected in brine (Sanchez et al., 2000), is due to the activity of pectinolytic enzymes and is not related to the yeast metabolism. The presence of these enzymes in the drupes is favored by an improper handling of the raw material and by poor fermentation conditions (Panagou and Tassou, 2006).

As far as the organoleptic features of olives is concerned, yeasts show other desirable metabolic properties such as esterase and lipolytic activities, both of which are strain-specific. The former has frequently been detected, while the isolation of strains with lipolytic activities has been reported to a lesser extent (Bautista-Gallego et al., 2011). Esterase positive yeasts are desirable because they can improve the flavor of olives through the formation of esters from free fatty acids. Strong lipase activity has been detected for *Candida boidinii*, *D. hansenii*, and *Torulaspora delbrueckii* while weak activity has been reported for *Pichia membranifaciens*; all the isolates from table olives have been *in vitro* tested (Psani and Kotzekidou, 2006; Rodríguez-Gómez et al., 2010). These authors have emphasized that a change in the free fatty acids composition of the olives is markedly higher in the presence of yeast populations than in sterile conditions, indicating that the lipases produced by these microorganisms modify the characteristics of the fat in the fruit and therefore also their organoleptic characteristics.

## MOLECULAR APPROACHES TO STUDY TABLE OLIVE FERMENTATION

Molecular methods have revolutionized modern approaches used to study the microbial populations and dynamics of fermented foodstuffs. Currently, two main approaches are adopted to investigate this aspect. A culture-dependent strategy, in which isolates are obtained from the food matrix by means of traditional microbiological methods and are subsequently studied by means of molecular analysis, and, more recently, methods called culture-independent, in which DNA or RNA are extracted directly and analyzed from the food matrix. The latter allows the complexity and evolution of the microbial ecology to be understood more easily during food fermentation since they detect all the populations, even the not culturable ones (VBNC). Furthermore, the populations that are in stressed or injured states can also be detected (Cocolin et al., 2011). These culture-independent methods are not so useful for the final selection of new starter strains, but they are of fundamental importance to have a complete overview of the food ecology and an exact definition of the main microbial species involved in fermentation process. In the case of table olive, which are still often produced by means of uncontrolled fermentation, the comparison between the results of culture-dependent and -independent ecological studies are important to highlight the main species of LAB and yeasts involved in the fermentation and hence those that are the most suitable for use as starter cultures.

## CULTURE-DEPENDENT APPROACH APPLIED TO TABLE OLIVE FERMENTATION

The two most representative species of LAB ecology in table olives (*L. plantarum* and *L. pentosus*) are genotypically closely related: the sequencing of the 16S rRNA fragment cannot discriminate between them (De Bellis et al., 2010) and at the same time they show highly similar phenotypes. Nevertheless, in some ecological studies (Randazzo et al., 2004; Bevilacqua et al., 2010), an initial distinction has been made between strains isolated from different olive fermentations by means of miniaturized identification systems, such as API (BioMérieux), which is based on carbohydrate fermentation patterns. Although the application of phenotypic techniques has proved to be useful for certain LAB, there is a general awareness that similar phenotypes displayed by strains do not always correspond to similar or even closely related genotypes (Temmerman et al., 2004). In fact, in these studies, the initial identification, by means of metabolic pathway profiles, was subsequently verified with genotypic methods and the comparison did not often give unequivocal results. As confirmation of this, Randazzo et al. (2004) showed that strains initially identified as *L.plantarum* by the API system were later assigned to the *L. brevis* species through molecular analysis. As the authors reported, this fact could be due to the very similar patterns of the fermented carbohydrates of the two facultative heterofermentative species which only different as far as the melibiose and raffinose fermentation are concerned.

With reference the molecular approach, Berthier and Ehrlich (1998) proposed the amplification, by PCR, of a long fragment of the spacer region interposed between the 16S rRNA and 23S rRNA genes. Species specific PCR allowed the closely related species *L. plantarum*, *L. pentosus*, *L. paraplantarum*, *L. curvatus*, *L. graminis*, and *L. sake* to be discriminated using a single forward primer and different reverse primers. However, this method does not concurrently discriminate between *L. plantarum*, *L. pentosus*, and *L. paraplantarum*, therefore it does not seem to be the best solution for the ecological study of table olives. Torriani et al. (2001) developed an interesting multiplex PCR assay, based on the amplification of the *recA* gene sequence, which performed a simultaneous distinction of these three lactobacilli. The amplicons were 318, 218, and 107 bp in size for *L. plantarum*, *L. pentosus*, and *L.paraplantarum*, respectively. This strategy leads to the unambiguous identification of strains belonging to these species in a single reaction and is widely used to speciate isolates of the *L. plantarum* group. Many authors have recently used this rapid method to study microbial ecology in both green and black table olives since it allows an immediate direct screening of the total lactobacilli isolated population (Franzetti et al., 2011; Doulgeraki et al., 2012). Initial discrimination by means of different PCR methods was instead used in other studies (Hurtado et al., 2008; De Bellis et al., 2010). Hurtado et al. (2008), for instance, performed an internal transcribed spacer (ITS)-PCR followed by restriction fragment length polymorphism (RFLP) enzymatic digestion of a 16S rRNA gene fragment according to Rodas et al. (2003). The subsequent identification was based on a comparison of the isolate band profile with the patterns obtained from type culture strains and it did not lead to the differentiation of the strains belonging to the *L. plantarum* group, and therefore had to be followed by *rec*A gene multiplex PCR.

In a recent work, carried out on Spanish style table olive fermentations the presence of an autochthonous starter culture during the process was monitored by combining Rep-PCR, RAPD-PCR, and restriction analysis of the *hsp*60 gene (Aponte et al., 2012). Starting from an unified cluster analysis (RAPD and rep-PCR profiles grouped together) of isolates, a discrimination of the species was performed. The final identification of representative strains of each pattern group was obtained after a restriction analysis of a 499-bp DNA fragment belonging to the *hsp*60 gene, which was conducted accordingly to the procedure described by Blaiotta et al. (2008). This is an alternative method to the PCR proposed by Torriani et al. (2001). Briefly, this PCR-RFLP assay is carried out using two endonuclease *Alu*I and *Taq*I (in separate reactions) which can lead to the identification and differentiation of 43 species of *Lactobacillus*, including *L. paraplantarum*, but it excludes the *L. plantarum*/*L. pentosus* pair which can be differentiated by a further restriction analysis with *Sau*3AI or *Mse*I. However, it should be emphasized that this second restriction assay did not lead to an unequivocal differentiation between the strains belonging to the *L. plantarum* and *L.arizonensis* species. A brief summary of the studies recently performed with the previously described molecular methods, of the main LAB species found and of the isolation matrices is reported in **Table 1**.

**Table 1:** Recent culture-dependent molecular approaches used to identify the LAB population in different types of table olives.

References	Matrices	Molecular approaches	Country	Main species
Randazzo et al. (2004)	Untreated green olives	RFLP-PCR of 16s rDNA and comparison with the profile of known species	Italy	*L. casei, L. brevis*
De Bellis et al. (2010)	Spanish style green olives	Rep-PCR followed by cluster analysis and sequencing of the most representative strains. Multiplex PCR (Torriani et al., 2001) for the *L. plantarum* group	Italy	*L. pentosus, Leuconostoc mesenteroides*
Bevilacqua et al. (2010)	Spanish style green olives	ITS-PCR followed by cluster analysis and sequencing of the most representative strains	Italy	*L. plantarum*
Franzetti et al. (2011)	Marketed olives	16S rRNA gene amplification and Multiplex PCR (Torriani et al., 2001) for the *L. plantarum* group	Italy	*Pediococcus acidilactici, L. pentosus, Leuconostoc mesenteroides*
Aponte et al. (2012)	Spanish style green olives	Combination of Rep-PCR, RAPD-PCR, and restriction analysis of the *hsp60* gene (Blaiotta et al., 2008)	Italy	*L. coryniformis, L. plantarum, L. pentosus*
Hurtado et al. (2008)	Untreated green olives	RFLP-PCR of 16s rDNA. Multiplex PCR (Torriani et al.,2001) for the *L. plantarum* group	Spain	*L. plantarum,L. paraplantarum*
Doulgeraki et al. (2012)	Greek style black olives	PFGE and sequencing of the most representative strains. Multiplex PCR (Torriani et al., 2001) for the *L. plantarum* group	Greece	*L. pentosus, L. plantarum, Pediococcus ethanolidurans*

As for yeasts, the restriction analysis of the ITSs (ITS1 and ITS2) and the 5.8S rRNA gene described by Esteve-Zarzoso et al. (1999) has been used to identify a total of 132 species belonging to 25 different genera, including teleomorphic and anamorphic ascomycetous and basidiomycetous yeasts. In many cases, the size of the PCR products and the restriction patterns obtained with the *Cfo*I, *Hin*fI, and *Hae*III endonucleases produced a unique profile for each species; for this reason, this rapid molecular method is used frequently in most ecology studies that target yeast diversity. Several studies have demonstrated its suitability for the identification of the heterogeneous yeast populations present in table olives. In addition, restriction analysis with the *Msp*I endonuclease can be performed for *S. cerevisiae* to discriminate between *S. cerevisiae* and *S. paradoxus* (Fernández-Espinar et al., 2000). An initial identification through a comparison of the restriction profile allows different species to be grouped while the final identification of isolates is usually confirmed by sequencing (domain D1/D2) of a representative strain for each group (Hurtado et al., 2008; Nisiotou et al., 2010; Muccilli et al., 2011). The main studies on table olive microbiota in the last few years have been performed with this molecular approach. Overall the *Candida*, *Pichia*, and *Saccharomyces* genera resulted to dominate the fermentation process (Hurtado et al., 2008; Aponte et al., 2010; Nisiotou et al., 2010; Bautista-Gallego et al., 2011; Franzetti et al., 2011; Alves et al., 2012).

As far as the typing of LAB strains and less frequently of yeasts is concerned, different methods have been described in literature for numerous types of fermented food (Iacumin et al., 2006; Zamfir et al., 2006; Van Hoorde et al., 2008; Sengun et al., 2009; Pathania et al., 2010). The most commonly used methods for the molecular typing of LAB isolated from table olives are rep-PCR and RAPD-PCR (Bevilacqua et al., 2010; De Bellis et al., 2010; Hurtado et al., 2010; Aponte et al., 2012). It should be noted that, in most cases, the molecular typing has been used to group the isolates from olive fermentations in relation to their genetic homology and then to choose the representative strains, which are identified using the previously described molecular techniques (Torriani et al., 2001; Blaiotta et al., 2008). As for the results of the characterizations, Aponte et al. (2012) combining the profiles obtained from the two typing techniques in a single dendrogram, detected a high similarity (97% according to the Pearson correlation index) between the lactobacilli strains isolated from Spanish style green olives in each fermentation stage. The same authors noted that RAPD-PCR recognized a greater number of homologous profiles than the rep-PCR technique. The latter method was used by De Bellis et al. (2010) and Hurtado et al. (2010) to follow the growth dynamics and the effective presence of inoculated starter cultures using one probiotic strain of *L. plantarum* and strains of *L. pentosus* inoculated singly or combined with yeasts, respectively, as a starter. In the first case De Bellis et al. (2010) performed five Spanish style fermentations at different temperatures and with different salt concentrations in the brines and they highlighted a high biodiversity on the olive surface in all the trials, especially at room temperature at which they recorded the highest heterogeneity (expressed according to the Shannon diversity index). In this work, in addition to the inoculated starter, LAB strains isolated belonged to the *L. pentosus* species in fermentations performed with 8% of NaCl and *Leuconostoc mesenteroides* when the brine were less concentrate. *L. pentosus* was also the most representative species in the study by Hurtado et al. (2010) conducted on natural green olives (without debittering), although they found a lower strain biodiversity than De Bellis et al. (2010). Unlike the three previous works, Bevilacqua et al. (2010) only conducted a typing of LAB strains isolated from Spanish style green olives after their identification. The results have shown a relatively low biodiversity between the *L. plantarum* strains, the main species involved in this fermentation.

Again with reference to molecular typing, Doulgeraki et al. (2012) exploited the potential of pulsed field gel electrophoresis (PFGE), both for the characterization of LAB and the yeast population present on the surface of black olives packed in pouches with different gas compositions and storage times. As far as the LAB are concerned, the two main parts of the isolated strains belonging to the *L. plantarum* and *L. pentosus* species and the molecular analysis at the strain level have revealed a great genetic diversity of the isolates, especially between the *L. pentosus* strains. Instead, the PFGE analysis of isolated yeasts has shown a low biodiversity in this population since the cluster analysis only highlighted the presence of five species, with internal heterogeneity.

## DIRECT PROFILING OF MICROBIAL ECOLOGY IN TABLE OLIVE FERMENTATION

Numerous culture-independent studies on vegetable fermentations can be found in the literature (Kim and Chun, 2005; Lee et al., 2005; Nakayama et al., 2007; Endo et al., 2008), but relatively few on table olives (Ercolini et al., 2006; Chamkha et al., 2008; Aponte et al., 2010; Abriouel et al., 2011).

Recently, a complete direct profiling of the total microbiota and the specific yeast dynamics of two different green table olives has been performed using denaturing gradient gel electrophoresis (DGGE; Abriouel et al., 2011; Muccilli et al., 2011). In brief, this culture-independent method has been applied extensively in various research areas and it is based on the electrophoresis separation of double stranded PCR products in a polyacrylamide gel containing a gradient of chemical denaturants (urea and formamide). The migration of each DNA fragment is not related to its size, but it is separated in function of its specific denaturing profile. Double stranded DNA becomes partially single stranded when it is subjected to an increase in the denaturing environment and in relation to its G + C % (Temmerman et al., 2004; Cocolin et al., 2011).

The first study was conducted by Abriouel et al. (2011) on an industrial fermentation process of *Aloreña* table olives, carried out without any prior lye treatment in different environmental conditions (temperature and fermenters). The authors considered the dynamics of the *Bacteria*, *Archaea*, and eukaryotes (yeasts and molds) present in the brine during the fermentation using the protocols proposed by Ogier et al. (2002), Casamayor et al. (2000), and Cocolin et al. (2000), respectively. This study did not confirm the usual bacterial profile detected in other green table olive fermentations, in which *L. plantarum* and *L. pentosus* are the predominant species. In this case the ecology was more complex and pointed out the presence of *L. paracollinoides* and *L.vaccinostercus*/*L. suebicus*, especially at the end of fermentations carried out at ambient temperature. Vice versa, the absence of LAB strains in the cold fermentation trial confirmed data already available in the literature. In these cases LAB were not able to compete against other microorganisms (especially Gram-negative bacteria) due to the low quantity of sugars available in the brines (Tassou et al., 2002; Lopez et al., 2005; Arroyo-López et al., 2008). The direct approach used in this study made it possible to detect some Gram-negative bacteria in brines, such as *Thalassomonas agarivorans*, non-fermentative bacteria, that has never before been isolated in table olives. This organism is ubiquitous in coastal water and it was most probably introduced in the brines through the marine salt used in their preparation. The presence of marine bacteria in brines has also been reported by Chamkha et al. (2008) for Tunisian black olives. This result was obtained by means of a culture-independent investigation on microbial ecology using PCR-single strand conformation polymorphism (SSCP) analysis, according to Delbes et al. (2001). Other studies have also reported the identification of Gram-negative bacteria in brines. Abriouel et al. (2011) detected *Pseudomonas *spp. together with *Sphingomonas* sp./*Sphingobium* sp./*Sphingopyxis* sp. throughout the whole fermentation process. The presence of *Pseudomonas* spp. has only been reported using the culture-dependent method on the surface of black olives and in brine in the early fermentation stages (Nychas et al., 2002; Ercolini et al., 2006). Its occurrence may play a negative role on the stability and the safety of table olives due to both the direct reduction in final acidity of the brine and to the indirectly production of biogenic amines.

The study carried out by Muccilli et al. (2011) using the DGGE approach focused on the yeast population dynamics in brine during the fermentation of directly brined Sicilian green table olives. At the same time, the authors performed a culture-dependent yeast ecology investigation according to the method proposed by Esteve-Zarzoso et al. (1999), and which is described above. In this context, the direct profiling results showed a lower biodiversity in the yeast population than in the parallel results obtained from the culture-dependent study. The direct profiling study emphasized the presence of only five distinct species belonging to four genera, while seven different species of yeasts were identified using the dependent culture approach. The authors mainly connected this occurrence to the complexity of the matrices (olive brines) which can lead to a low DNA yield and a more difficult PCR reaction. Furthermore, the initial template DNA ratio and template competition may affect the detection of microorganisms present in complex microbiota. However, another study of microbiota ecology carried out on untreated green olive fermentations has shown a higher number of species identified using direct profiling than when a culture-dependent approach was adopted (Aponte et al., 2010). It should be pointed out that PCR-DGGE was not used in the aforementioned study, but RFLP-PCR was applied directly to DNA extracted from the matrices.

The lower microbial diversity detected in PCR-DGGE with respect to that found by means of isolation and identification of the yeast strains has already been demonstrated by Di Maro et al. (2007) in wine fermentations. However, this culture-independent approach has allowed the presence of *I. orientalis* to be discovered for the first time in the late stages of fermentation (Muccilli et al., 2011). As already mentioned, this species is common in table olives, but had not been previously detected at the end of fermentation processes (Doulgeraki et al., 2012), probably due to its low competitiveness in a culture medium or to the physiological state of its cells.

A different direct approach from the previous ones has been attempted by Ercolini et al. (2006) using fluorescence *in situ* hybridization (FISH) with a 16S rRNA gene probe in order to detect the presence of *L. plantarum* on the olive surfaces. The main advantage of the FISH is that it offers the possibility of detecting the microorganisms directly in their habitats without culture-dependent isolation of the cells or culture-independent extraction of the nucleic acids prior to the identification. However, no hybridization signal was detected in this study in at least 30 fields of observation when the specific probe for the *L.plantarum* group was used, probably because of the low sensitivity of the FISH. However, even though this method could be applied, it would only allow a specific group of species be detected and it would not allow a complete direct ecology study to be conducted.

## CONCLUSIONS AND FUTURE PROSPECTS

As previously mentioned, in relation to culture-dependent studies of olive LAB microbiota, the initial screening of isolates by means of the multiplex PCR method described by Torriani et al. (2001) seems to be an optimal approach. This method permits all the strains belonging to the *L. plantarum* group, the most representative lactobacilli species of table olive microbiota to be identified quickly. All the isolates that do not belong to this group, and which usually represent a minority of the LAB population, can then be identified through sequencing. A far as yeasts are concerned, most culture-dependent studies have been performed using the method developed by Esteve-Zarzoso et al. (1999). The accuracy of this ITS-PCR-RFLP assay allows the heterogeneous microbiota present on the surface of olives and in brines to be detected quickly. Although it is true that a great deal of knowledge has been obtained in the study of table olive microbiota, few studies have gone beyond the identification stage of the species in order to highlight the biodiversity of the isolated strains. So far, only one study present in literature has performed a final molecular characterization of the isolates (Doulgeraki et al., 2012).

As far as direct profiling is concerned, PCR-DGGE has emerged, over the last 20 years, as a promising method to define the microbial ecology of food and beverage ecosystems, but, as reported in the studies described above, it offers a limited capacity to discriminate between species with very similar 16S rRNA gene sequences, such as *L. plantarum* and *L. pentosus*, species which play an important role in table olive microbial ecology. A few examples of different culture-independent approaches, from DGGE, which have been applied to table olive fermentation, are available in the literature, but they do not seem to show a better discriminating ability (Chamkha et al., 2008). A potential solution to direct profiling studies of table olive ecology could involve PCR-DGGE

targeted to the *rpo*B gene. as described by Dahllof et al. (2000) and applied by Rantsiou et al. (2004) to follow LAB dynamics in foodstuffs. Furthermore, it should be noted that in the studies carried out on table olives using PCR-DGGE, no one has focused attention on the RNA target. Its study would allow the LAB and yeasts that are metabolically active during the fermentations to be identified.

In the near future, meta-analysis methods, such as metagenomics and metatranscriptomics, will allow the diversity and activity of microorganisms in olive fermentations to be studies in depth through the application of next generation sequencing. These approaches have already been applied to other kinds of fermented foods and their potentials has been demonstrated (Humblot and Guyot, 2009).

## Conflict of Interest Statement

The authors declare that the research was conducted in the absence of any commercial or financial relationships that could be construed as a potential conflict of interest.
